# Supplementary material for: Induction of Glucoraphasatin Biosynthesis Genes by MYB29 in Radish (Raphanus sativus L.) Roots
Source: Int J Mol Sci. 2020 Aug 10;21(16):5721. doi: 10.3390/ijms21165721 (PMC7461053; doi:10.3390/ijms21165721)
Supplement: Supplementary file 1 [file ijms-21-05721-s001.zip › Supplementary Figure.pdf]

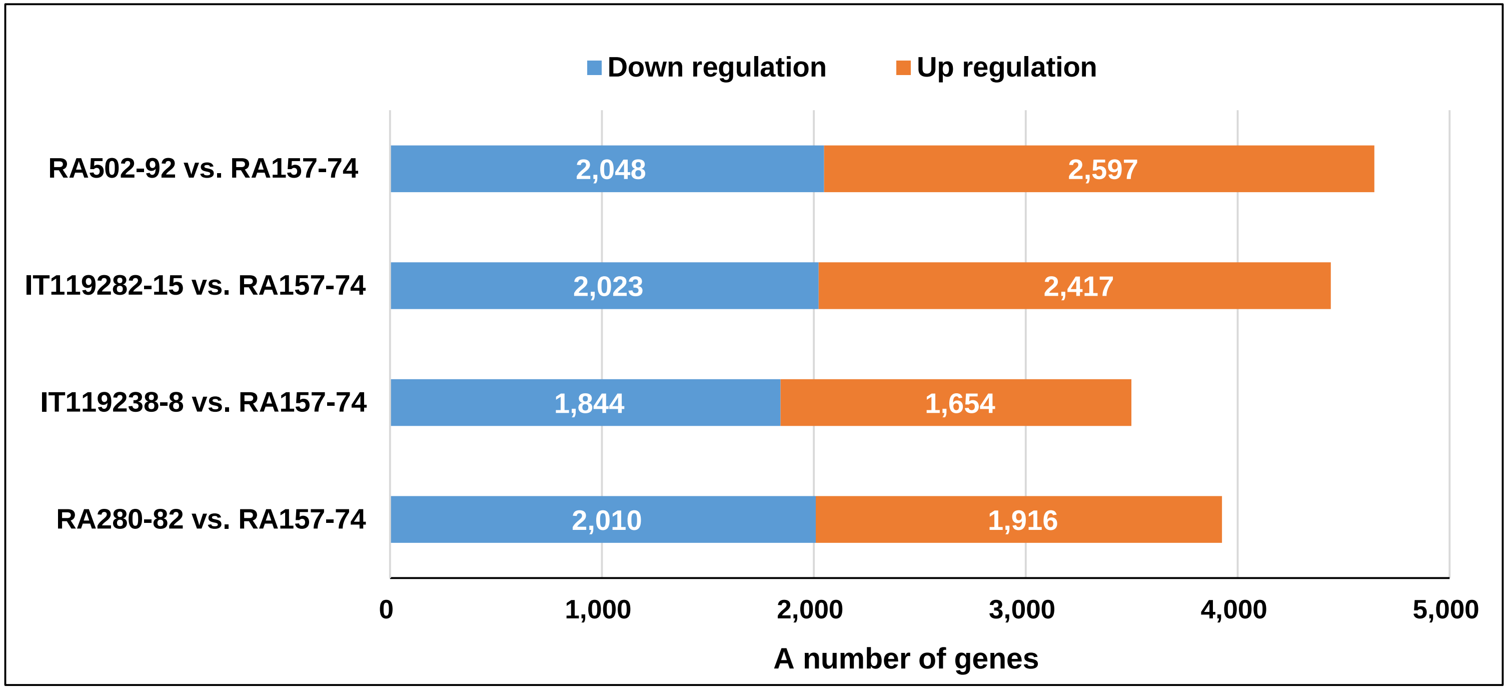

Figure S1. A number of differentially expressed genes (DEGs) in the RA157-74 roots compared with the RA502-92, IT119238-15, IT119238-8, and RA280-82 roots.

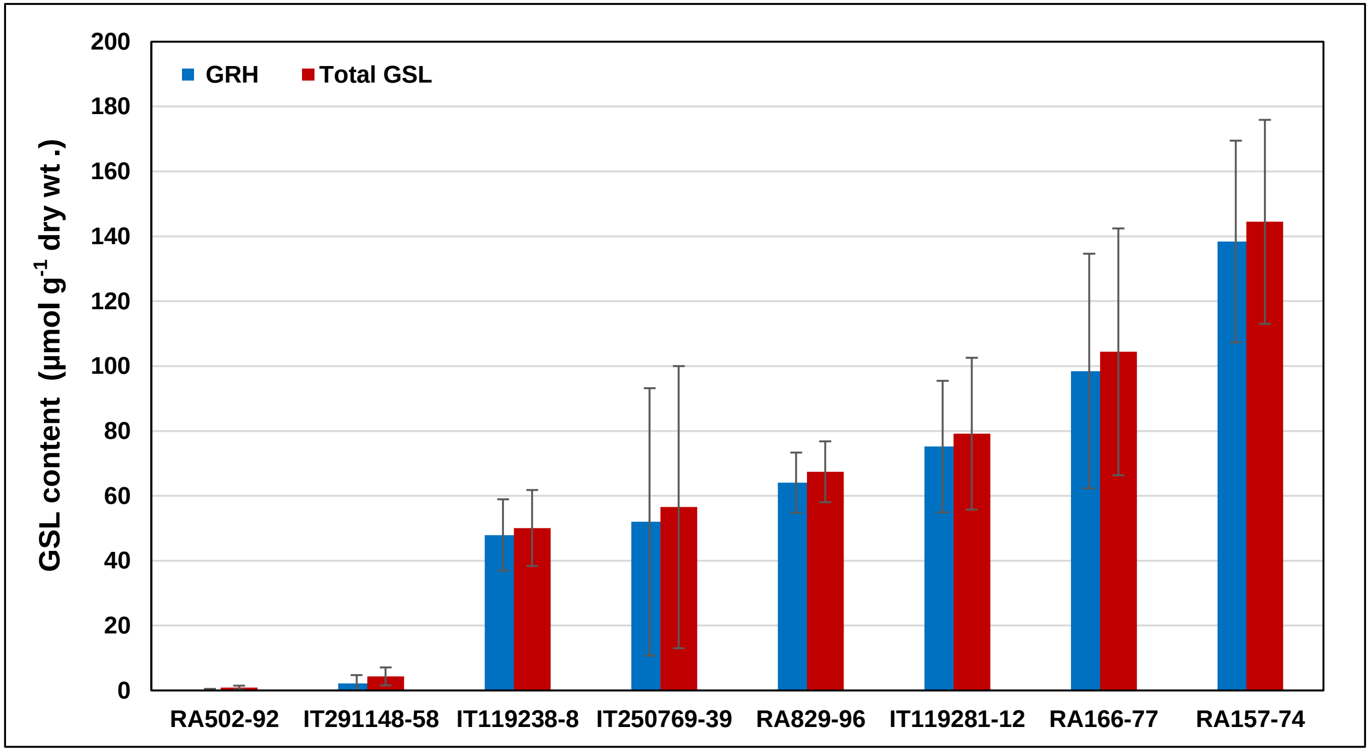

Figure S2. Total GSL and GRH content of eight individual accessions. The blue and red columns represent the GRH and total GSL, respectively. The error bars represent the SD (standard deviation) in three replicate experiments.

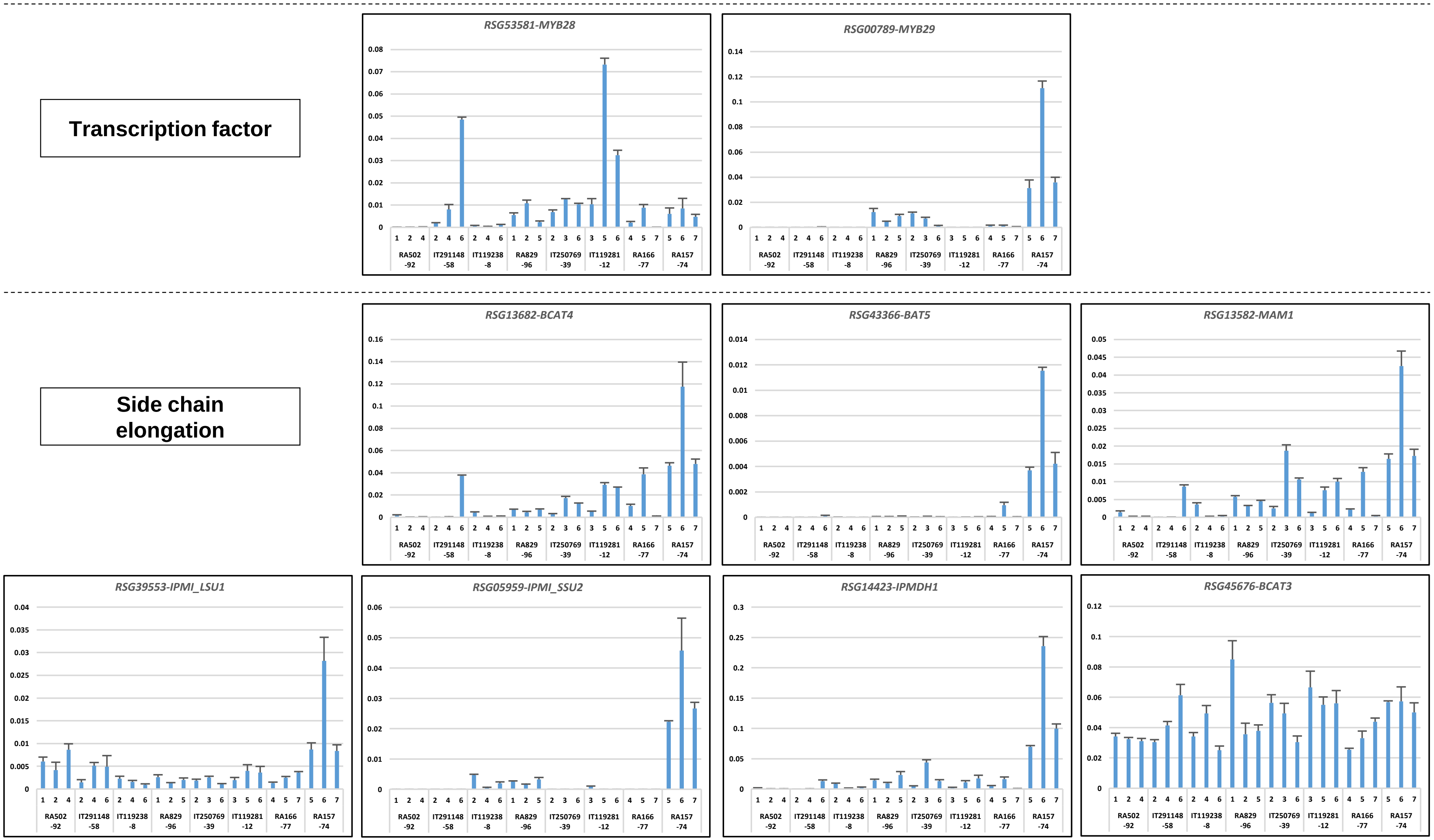

Figure S3. qRT-PCR analysis of aliphatic GSL biosynthesis genes in the radish roots. Expressions of the transcription factors and genes in the chain elongation step were analyzed by qRT-PCR using the delta CT method. The error bars represent the SD (standard deviation) in three replicate experiments.

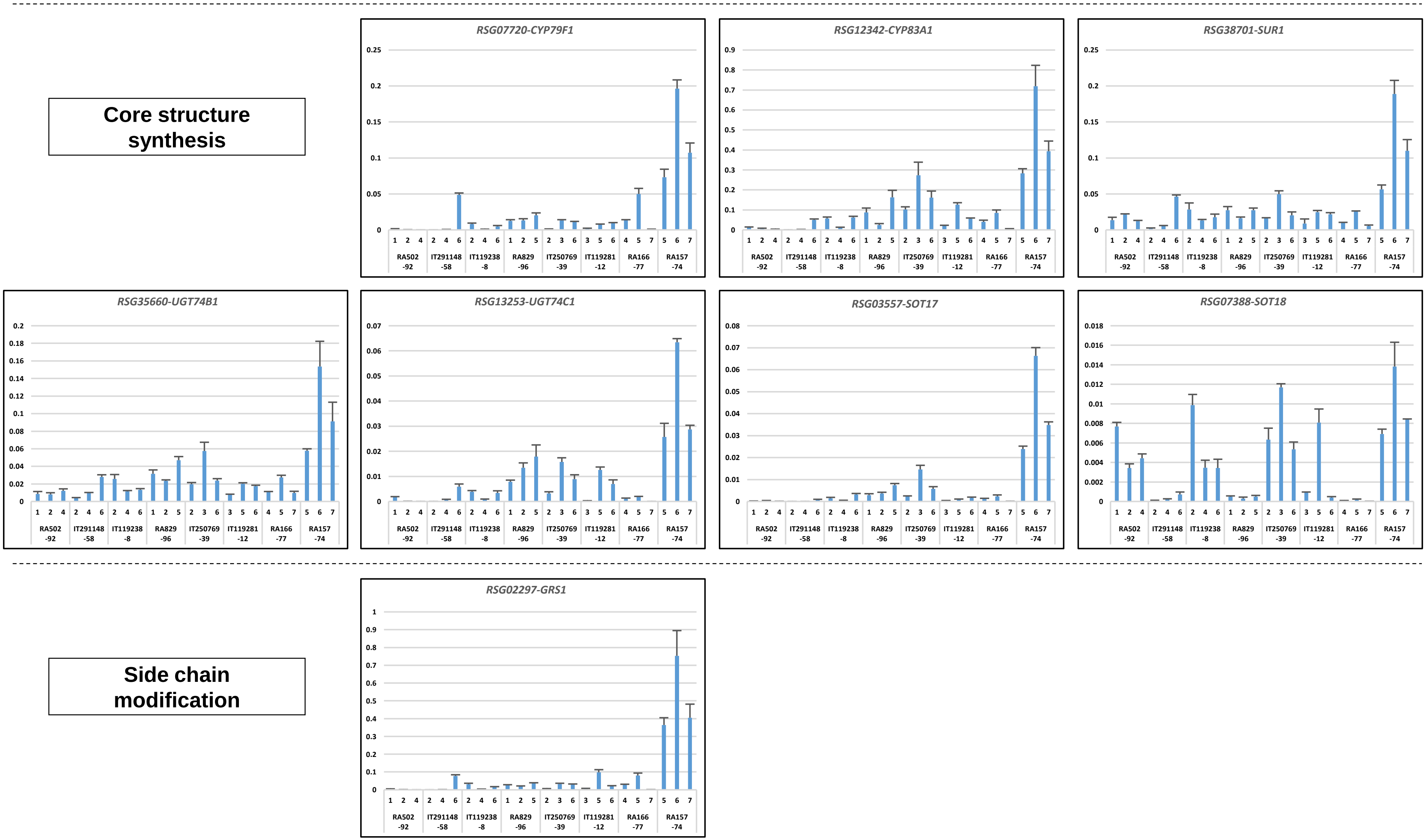

Figure S4. qRT-PCR analysis of aliphatic GSL biosynthesis genes in the radish roots. Genes expressions involved in core structure synthesis and side chain modification were analyzed by qRT-PCR using the delta CT method. The error bars represent the SD (standard deviation) of three replicate experiments.

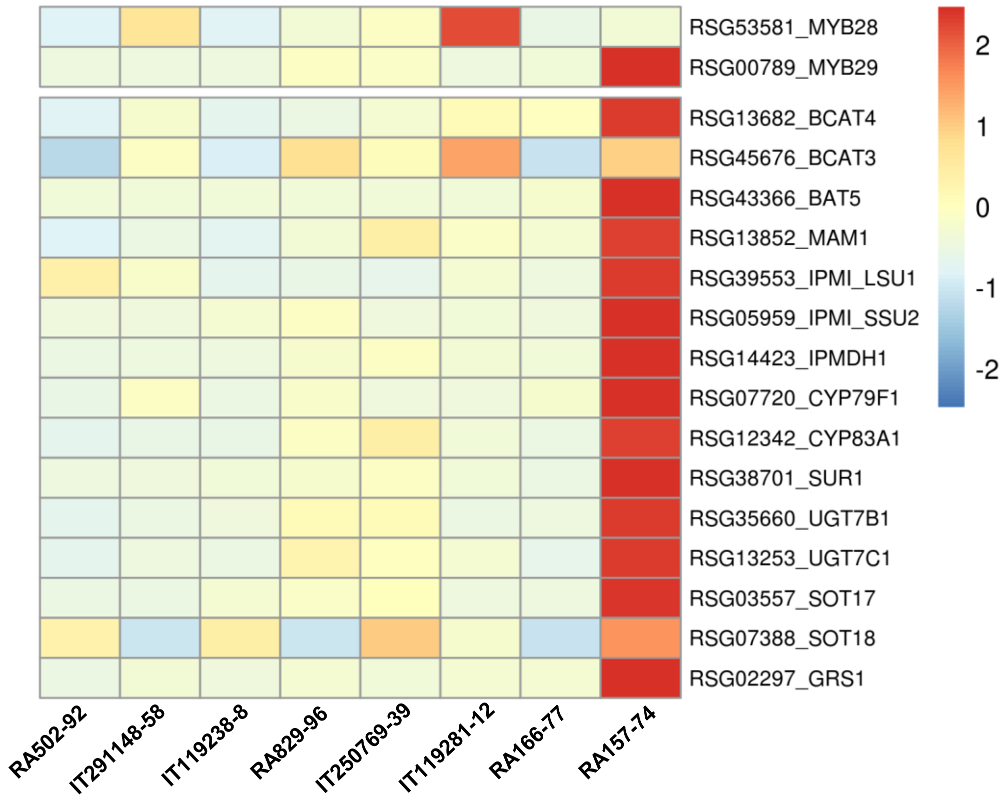

Figure S5. Analysis of DEGs involved in the GRH biosynthesis. Genes expressions were analyzed by qRT-PCR using the delta CT method. Expression values of individual genes were scaled per row (i.e. per gene) for visualization of expression peaks of genes among the accessions.
